# Associations between exposure to sexual abuse, substance use, adverse health outcomes, and use of youth health services among Norwegian adolescents

**DOI:** 10.1186/s12889-023-16261-y

**Published:** 2023-07-11

**Authors:** TH Stea, AM Steigen, CR Dangmann, MD Granrud, T Bonsaksen

**Affiliations:** 1 Department of Health and Nursing Science, University of Agder, Kristiansand, Norway; 2 Department of Health and Nursing Sciences, Inland Norway University of Applied Sciences, Elverum, Norway; 3 Department of Social Sciences and Guidance, Faculty of Social and Health Science, Inland Norway University of Applied Sciences, Elverum, Norway; 4 Department of Health, Faculty of Health Studies, VID Specialized University, Stavanger, Norway

**Keywords:** Adolescents, Sexual abuse, Substance use, Depressive symptoms, Headache, Medication use, Self-harm, Suicidal thoughts, Suicide attempts, Youth health services

## Abstract

**Background:**

A strong association between sexual abuse and adverse health outcomes has been reported among adolescents. The present study aimed to provide more information about adverse health outcomes associated with sexual abuse and substance use, and to examine the use of youth health services among Norwegian adolescents.

**Methods:**

National representative cross-sectional study among 16–19-year-old Norwegian adolescents (n = 9784). Multivariable regression analyses, adjusted for socioeconomic status and age, were used to examine the association between exposure to sexual abuse, substance use and health risk factors, and the use of youth health services.

**Results:**

Adolescents exposed to sexual abuse had higher odds of depressive symptoms (males: OR:3.8; 95% CI:2.5–5.8, females: 2.9;2.4–3.5), daily headache (males: 5.3;2.8–10.1, females:1.9; 1.5–2.4), high medication use (males: 3.2;1.7-6.0, females: 2.0;1.6–2.6), self-harm (males: 3.8;2.4-6.0, females:3.2; 2.6–3.9), suicidal thoughts (males: 3.3; 2.2-5.0, females:3.0; 2.5–3.6) and suicide attempts (males: 9.5;5.6–16.0, females:3.6;2.7–4.9). Furthermore, exposure to sexual abuse was associated with higher odds of using school health services (males: 3.9;2.6–5.9, females: 1.6;1.3–1.9) and health services for youth (males: 4.8;3.1–7.6, females: 2.1;1.7–2.5). In general, substance use was associated with increased odds of adverse health related outcomes and use of youth health services, but the strength of the relationships varied according to sex. Finally, results indicated a significant interaction between sexual abuse and smoking that was associated with increased odds of having suicidal thoughts for males (2.6;1.1–6.5) but a decreased odds of having suicidal thoughts and have conducted suicide attempts once or more for females (0.6;0.4-1.0 and 0.5;0.3–0.9, respectively).

**Conclusions:**

The present study confirmed a strong relationship between exposure to sexual abuse and health risks, especially among males. Moreover, males exposed to sexual abuse were much more likely to use youth health services compared to sexually abused females. Substance use was also associated with adverse health outcomes and use of youth health services, and interactions between sexual abuse and smoking seemed to influence risk of suicidal thoughts and attempts differently according to sex. Results from this study increase knowledge about possible health related effects of sexual abuse which should be used to identify victims and provide targeted treatment by youth health services.

**Supplementary Information:**

The online version contains supplementary material available at 10.1186/s12889-023-16261-y.

## Background

Childhood sexual abuse (CSA) is a major public health problem associated with a wide range of adverse psychosocial and health outcomes [1, 2]. Moreover, evidence suggests that CSA has substantial economic and social costs on the societal level, including costs of health and child welfare services, and durable negative impact on human capital and economic wellbeing on the individual level [3–6].

CSA can be defined as an act that is both abusive and sexual in nature, to which a child under 18 years of age cannot consent [7], and has been identified as a globally widespread problem [8]. However, prevalence rates of CSA vary between continents with lower rates in Europe than Australia, Africa and North America [9] which may partly be explained by cultural and social differences in disclosure [10]. Methodological dissimilarities between studies conducted in the Nordic countries may also contribute to the large observed variation in prevalence of sexual abuse, with rates between 3 and 23% for males and 11–36% for females [11]. Moreover, an increased risk of abuse from early adolescence has been confirmed, and peer adolescents constitute the largest group of perpetrators [11, 12].

CSA is associated with a myriad of deleterious outcomes across the life span, especially mental, behavioral, sexual, and physical health problems, as well as psychosocial difficulties [1, 13–15]. Research has also shown that CSA is associated with increased risk of using legal and illicit substances, including alcohol, tobacco, and drugs [16–20]. Moreover, a growing body of evidence has also suggested a strong association between exposure to sexual abuse and risk of self-harm and suicide among adolescents [21–25]. Although exposure to sexual abuse has been identified as a single most important risk factor for self-harm among adolescents, evidence suggesting that co-occurrence of CSA with mental health problems and/or substance use, greatly increase the risk of self-harm and suicidal behavior [13, 26].

Prevalence of CSA and subsequent health consequences vary with the age and sex of the victim [27]. Meta-analyses have suggested a ratio of approximately 2.5 females for every one male victim of sexual abuse across continents [8, 28, 29]. Male victims of sexual abuse, however, have recently been identified as a steadily increasing phenomenon [30]. Moreover, male victims of sexual abuse have shown increased risk of suicidal behavior and attempts, even in the absence of depression, hopelessness, and family dysfunction, whereas the relationship between sexual abuse and suicidality among females seems to be mediated by depression, hopelessness, and family dysfunction [31]. Furthermore, adolescents who perceive their families to be less well off than others have been twice as likely to report sexual abuse as those of ample or medium family affluence, and the effect was stronger among females than males [32].

Researchers have concluded that it is essential be aware of the symptoms and consequences of CSA among both males and females in order to discover and provide support, appropriate care and treatment for the survivors [33]. However, the ability of health professionals employed at youth health services to provide appropriate support is contingent upon knowledge about the broad spectrum of symptoms and behaviors associated with CSA. Moreover, it relies on a nuanced understanding of how such associations vary between different groups of adolescents. Thus, the main aim of the present study was to examine the association between CSA and substance use and adverse health outcomes, including headache, use of non-prescription medication, depressive symptoms, self-harm, suicidal thoughts and behaviors, and use of youth health services. A secondary aim was to explore possible moderating effects of concurrent substance use on the associations between CSA, adverse health outcomes and use of youth health services.

## Methods

### Design and participants

The Ungdata surveys are conducted annually across most Norwegian municipalities and is an essential source of information on young peoples’ health, well-being, attitudes, and behaviors across a range of areas (see www.ungdata.no). Norwegian Social Research (NOVA) at Oslo Metropolitan University is responsible for the surveys in a collaboration with the Regional Drug and Alcohol Competence Centres (KoRus). The surveys are financed partially by the Norwegian Directorate of Health. The present study was based on results from the Ungdata survey conducted in 18 Norwegian municipalities, which in 2021 was completed by 9784 Norwegian high school students (grade 11–13, age range 16–19 years). Students decided in school whether they wanted to participate after being informed that participation was voluntary and that they could skip questions that they did not want to answer. The study was conducted as a web-based questionnaire administered at school during school hours with a teacher or an administrator present to answer questions. The participants used approximately 30–45 min to complete the questionnaire.

### Measures

All measures were based on adolescent self-reports.

#### Outcome variables

##### Depressive symptoms

Depressive symptoms were measured using a six-item scale, which is based on the Hopkins Symptom Checklist-90 [34]. The adolescents were asked whether, during the past week, they have been affected by any of the following: (1) “felt that everything is a struggle;” (2) “had sleep problems;” (3) “felt unhappy, sad, or depressed;” (4) “felt hopelessness about the future;” (5) “felt stiff or tense;” or (6) “worried too much about things.” Each item was rated based on four response alternatives: 1 = “not been affected at all;” 2 = “not been affected much;” 3 = “been affected quite a lot;” and 4 = “been affected a great deal.” Mean scores were computed, ranging from 1 to 4; high scores indicated high levels of depressive symptoms. To capture more serious depressive symptoms, a dichotomous variable of depressive symptoms was used, where the cut-off was set at 3.0 to classify participants into those with the average scores of quite distressed or higher [1] and those with lower scores (0). Previous studies have shown that the prevalence of adolescents scoring above this cut-off was within the range of prevalence rates of depressive disorders commonly found in adolescent community samples, including in Norway [35, 36]. Additionally, the scale has been psychometrically evaluated using previously collected data among Norwegian adolescents and has demonstrated good reliability (Person Separation Index: 0.802) and appears overall to work reasonably well on a general level [37].

##### Headache

The adolescents answered a question about whether they had experienced headache during the previous month with the following response options: “never,” “sometimes,” “often,” or “daily.” We dichotomized the response alternatives into the categories every day and less than every day [38].

##### Use of pain medication

The use of pain medication was assessed by asking respondents how often they had used nonprescription drugs (e.g., acetaminophen or ibuprofen) during the past month [39]. The question had five response alternatives: “never;” “less than once a week;” “once a week;” “more than once a week;” and “every day.” These response alternatives were dichotomized into the categories of every day (high medication use; reference category) and less than every day.

##### Self-harm behaviors

Information about exposure to self-directed violence was retrieved by asking the following questions: “Have you ever tried to harm yourself?”, “Have you ever thought about taking your own life?” Have you ever tried to take your own life?” Response alternatives were “no”, “yes, once” and “yes, several times”. Responses were dichotomized into “never” and “at least once” [40].

##### Use of health services for adolescents

In Norway, the school health service is available for all elementary-, junior- and senior- high school students during school hours, whereas youth health services are available in the local community after school-hours. Both primary services are free-of-charge, do not require referrals, and seek to promote mental and physical health, good social and environmental conditions, prevent diseases and injuries, reduce social inequalities in health, and prevent and uncover violence, abuse and neglect. Moreover, both these services shall identify children and adolescents at risk as early as possible, offer them services and refer them to other services if necessary [41]. In the present study, participants were asked whether they during the last 12 months had used the school health services and the youth health services, respectively. Both questions had four response alternatives: “never;” “1–2 times”, 3–5 times”, “6 times or more”. For the analysis, these responses were dichotomized into “never” and “at least once.”

#### Explanatory variables

##### Sexual abuse

Sexual abuse was assessed with the following question: “Has someone pressured or forced you to have intercourse or other sexual acts?”. Response options were never, once, 2–5 times and 6 or more times. For subsequent analyses, the results were dichotomized to distinguish between participants who had not been exposed to sexual abuse and those who had been exposed.

##### Adolescent substance use

Intoxication with alcohol was measured by asking respondents how many times they had been clearly intoxicated the past 12 months with five response alternatives: never, once, 2–5 times, 6–10 times, and > 11 times [42]. The variable was categorized as a binary variable including any alcohol intoxication episodes vs. no intoxication episodes.

Smoking (tobacco) was measured by asking respondents whether they smoke (Post et al., 2005). There were five response categories: have never smoked, have smoked earlier but quit, smoke more seldom than once a week, weekly smoking, but not daily, and daily smoking. This variable was applied as a binary variable including current smoking vs. no current smoking [43].

Cannabis use was measured by asking respondents how many times they had used hashish/marihuana/cannabis during the past 12 months with five response alternatives: never, once, 2–5 times, 6–10 times, and > 11 times. The variable was categorized as a binary variable including any use vs. no use [44].

#### Control variables

The Family Affluence Scale (FAS), version II, was applied as a measure of socioeconomic status [45], and this information was retrieved by asking respondents the following four questions: 1 “Does your family have a car?” with the following response options; no, yes one, and yes two or more; 2 “Do you have your own bedroom?” with response options: yes, and no; 3 “How many times have you travelled somewhere on holiday with your family over the past year?” with response options: never, once, twice, and more than twice; and 4 “How many computers or tablet computers does your family have?” with response options: none, one, two, and more than two. A sum score based on all four items was calculated and used as an overall measure of family affluence, with scores ranging between 4 (lowest affluence) and 13 (highest affluence). FAS II has proven to be a valuable tool for adolescent health studies as a study from 35 European and North American countries and regions, showed that country FAS II levels correspond well with national wealth indicators, indicating good criterion validity, and is strongly associated with a number of individual level health behaviors and outcomes [46].

Class-level was applied as a proxy for adolescent age and was measured by asking respondents which class-level they attended. Grade 11 equals the age range 16–17 years, grade 12 equals the age range 17–18 years, and grade 13 equals the age range 18–19 years.

Information about the participant’s sex was retrieved by asking participants in the Ungdata survey whether they were male or female.

### Data analysis

Descriptive analyses including all variables were performed. Logistic regression analyses were applied to explore possible associations between exposure to sexual abuse and substance use, any of the defined health-related outcomes, and use of health services for adolescents. Results were reported by odds ratios (ORs), 95% confidence intervals (CIs) with the respective p-values with a significance level set to p < 0.05. Multivariable analyses were adjusted for FAS and adolescent age. Interaction analysis was used to examine the influence of substance use on the strength of the relationship between exposure to sexual abuse and health related outcomes, including depressive symptoms, headache, use of medication, self-harm, suicidal thoughts and suicide attempts. Possible interaction effects were examined using LR tests (likelihood ratio tests) by contrasting models with and without interaction terms. The main effect model included the following variables: exposure to sexual abuse, alcohol intoxication, smoking, cannabis use, age and FAS as independent variables and was tested against models that included interactions between exposure to sexual abuse by different forms of substance use. In cases of statistically significant interaction terms, the analyses were repeated separately for each category of the moderator variable.

## Results

Descriptive data (Table 1) show that a higher number of females had experienced sexual abuse than males (10.9% vs. 2.3%). More males than females had frequently experienced alcohol intoxication (16.7% vs. 14.4%), were currently smokers (20.3% vs. 14.8%) and had used cannabis during the past 12 months (16.2% vs.10.3%). More females than males reported risk behaviors and adverse health effects, including depressive symptoms (29.7% vs. 11.8%), daily headache (12.3% vs. 3.0%), high medication use (9.3% vs. 3.0%), self-harm (19.8% vs. 9.2%), suicidal thoughts (28.9% vs. 19.5%) and suicide attempts (5.1% vs. 3.0%). A higher number of females than males had been in contact with school health services and health services for youth (41.6% vs. 15.8%, and 23.8% vs. 6.6%, respectively).

**Table 1:** Descriptive statistics among adolescent males (n = 4640) and females (n = 5144)

Variables		Males	Females	p-value*
**Control variables**	11th grade, high school, n (%)	1974 (43.6)	1980 (39.3)	< 0.01
	12th grade, high school, n (%)	1666 (36.8)	1678 (33.4)	
	13th grade, high school, n (%)	891 (19.7)	1374 (27.3)	
	Family Affluence Scale score, m (SD)**	9.8 (1.5)	9.8 (1.5)	0.092
**Exposure variables**	Sexual abuse, n (%)	106 (2.3)	554 (10.9)	< 0.01
	Alcohol intoxication last 12 months, n (%)	770 (16.7)	735 (14.4)	0.002
	Currently smoking, n (%)	940 (20.3)	760 (14.8)	< 0.01
	Cannabis use last 12 months, n (%)	742 (16.2)	528 (10.3)	< 0.01
**Dependent variables**	Depressive symptoms, n (%)	531 (11.8)	1498 (29.7)	< 0.01
	Daily headache, n (%)	120 (3.0)	556 (12.3)	< 0.01
	High medication use, n (%)	140 (3.0)	475 (9.3)	< 0.01
	Self-harm, n (%)	426 (9.2)	1017 (19.8)	< 0.01
	Suicidal thoughts, n (%)	900 (19.5)	1477 (28.9)	< 0.01
	Suicide attempts, n (%)	138 (3.0)	263 (5.1)	< 0.01
	School health service, n (%)	724 (15.8)	2114 (41.6)	< 0.01
	Health service for youth, n (%)	300 (6.6)	1208 (23.8)	< 0.01

*The chi-square test was used for categorical variables, and the independent-sample t test was used for continuous variables (Family Affluence Scale score)

**Higher scores on the Family Affluence Scale indicate more affluent

The results presented in Tables 2 and 3 show that those who had experienced sexual abuse had higher odds of depressive symptoms (males: OR:3.8; 95% CI:2.5–5.8, females: 2.9;2.4–3.5), daily headache (males: 5.3;2.8–10.1, females:1.9; 1.5–2.4), high medication use (males: 3.2;1.7-6.0, females: 2.0;1.6–2.6), self-harm (males: 3.8;2.4-6.0, females:3.2; 2.6–3.9), suicidal thoughts (males: 3.3; 2.2-5.0, females:3.0; 2.5–3.6) and suicide attempts (males: 9.5;5.6–16.0, females:3.6;2.7–4.9). Moreover, those who had experienced sexual abuse had higher odds of contacting school health services (males: 3.9;2.6–5.9, females: 1.6;1.3–1.9) and health services for youth (males: 4.8;3.1–7.6, females: 2.1;1.7–2.5) (Table 4).

**Table 2:** Modeled effects of sexual abuse and substance use on depressive symptoms, daily headache and use of medication

		Depressive symptoms		Daily headache		High medication use		
		Males *OR (95% CI)*	Females *OR (95% CI)*	Males *OR (95% CI)*	Females *OR (95% CI)*	Males *OR (95% CI)*	Females *OR (95% CI)*	
**Exposure variables**	Sexual abuse	3.8 (2.5–5.8)***	2.9 (2.4–3.5) ***	5.3 (2.8–10.1)***	1.9 (1.5–2.4)***	3.2 (1.7-6.0)***	2.0 (1.6–2.6)***	
	Alcohol intoxication	0.8 (0.6–1.1)	1.0 (0.8–1.2)	1.0 (0.6–1.8)	1.1 (0.8–1.4)	1.5 (1.0-2.4)	1.1 (0.9–1.5)	
	Smoking	1.2 (0.9–1.6)	1.7 (1.4-2.0)***	0.9 (0.5–1.5)	1.5 (1.1–1.9)**	1.5 (1.0-2.4)	1.6 (1.3–2.1)***	
	Cannabis use	2.0 (1.6–2.6)***	1.5 (1.2–1.9)***	1.8 (1.1–2.9)*	1.3 (1.0-1.7)	1.6 (1.0-2.5)*	1.2 (0.9–1.6)	
**Control variables**	FAS	0.8 (0.7–0.8)***	0.8 (0.8–0.8)***	0.9 (0.8-1.0)*	0.9 (0.8-1.0)**	0.9 (0.8-1.0)	1.0 (0.9–1.1)	
	Age	1.0 (0.9–1.2)	1.0 (0.9–1.1)	1.0 (0.8–1.3)	0.9 (0.8-1.0)**	0.8 (0.7-1.0)	0.9 (0.8-1.0)	

Note: *p < 0.05, ** p < 0.01, ***p < 0.001

**Table 3:** Modeled effects of sexual abuse and substance use on self-harm, suicidal thoughts and suicide attempts

		Self-harm		Suicidal thoughts		Suicide attempts		
		Males *OR (95% CI)*	Females *OR (95% CI)*	Males *OR (95% CI)*	Females *OR (95% CI)*	Males *OR (95% CI)*	Females *OR (95% CI)*	
**Exposure variables**	Sexual abuse	3.8 (2.4-6.0)***	3.2 (2.6–3.9)***	3.3 (2.2-5.0)***	3.0 (2.5–3.6)***	9.5 (5.6–16.0)***	3.6 (2.7–4.9)***	
	Alcohol intoxication	0.7 (0.5–0.9)*	1.0 (0.8–1.2)	0.9 (0.7–1.1)	1.0 (0.9–1.3)	0.9 (0.5–1.4)	1.2 (0.8–1.6)	
	Smoking	1.7 (1.3–2.3)***	2.3 (1.9–2.8)***	1.4 (1.1–1.7)**	2.0 (1.7–2.4)***	1.3 (0.8–2.1)	2.8 (2.1–3.9)***	
	Cannabis use	2.1 (1.6–2.8)***	1.9 (1.5–2.3)***	2.1 (1.7–2.6)***	1.9 (1.5–2.3)***	2.9 (1.9–4.6)***	2.0 (1.4–2.8)***	
**Control variables**	FAS (low)	0.8 (0.8–0.9)***	0.9 (0.8–0.9)***	0.9 (0.8–0.9)***	0.8 (0.8–0.9)***	0.8 (0.7–0.9)***	0.8 (0.7–0.9)***	
	Age	0.8 (0.7–0.9)**	0.6 (0.6–0.7)***	0.9 (0.8–0.9)**	0.7 (0.7–0.8)***	0.7 (0.5–0.9)**	0.6 (0.5–0.7)***	

Note: *p < 0.05, ** p < 0.01, ***p < 0.001

**Table 4:** Use of youth health services according to exposure to sexual abuse and substance use risk behavior

		School health service		Health service for youth		
		Males *OR (95% CI)*	Females *OR (95% CI)*	Males *OR (95% CI)*	Females *OR (95% CI)*	
**Exposure variables**	Sexual abuse	3.9 (2.6–5.9)***	1.6 (1.3–1.9)***	4.8 (3.1–7.6)***	2.1 (1.7–2.5)***	
	Alcohol intoxication	1.2 (1.0-1.5)	1.9 (1.7–2.2)***	1.6 (1.1–2.2)**	3.1 (2.6–3.7)***	
	Smoking	1.4 (1.1–1.7)**	1.3 (1.1–1.6)**	2.0 (1.5–2.7)***	1.2 (1.0-1.4)	
	Cannabis use	1.0 (0.8–1.3)	1.1 (0.9–1.4)	1.5 (1.1 2.1)**	1.7 (1.4–2.1)***	
**Control variables**	FAS (low)	0.9 (0.9-1.0)**	1.0 (1.0–1.0)	0.9 (0.8–0.9)**	1.0 (0.9-1.0)	
	Age	0.9 (0.8–1.1)	1.0 (0.9–1.1)	0.9 (0.7-1.0)	1.0 (0.9–1.1)	

Note: *p < 0.05, ** p < 0.01, ***p < 0.001

Frequent alcohol intoxication was associated with lower odds of self-harm among males (0.7;0.5–0.9) but not females, and higher odds of using school health services was found among females (1.9;1.7–2.2) but not males. Both males and females reporting frequent alcohol intoxication had increased odds of contacting health services for youth (males: 1.6;1.1–2.2, females: 3.1;2.6–3.7).

Cannabis use was also associated with higher odds of depressive symptoms (males: 2.0; 1.6–2.6, females: 1.5;1.2–1.9), daily headache among males (1.8;1.1–2.9), and high medication use among males (1.6;1.0-2.5), but not females. Moreover, cannabis use was associated with self-harm (males: 2.1;1.6–2.8, females:1.9;1.5–2.3), suicidal thoughts (males: 2.1; 1.7–2.6, females:1.9;1.5–2.3) and suicide attempts (males: 2.9;1.9–4.6, females:2.0;1.4–2.8). Being cannabis users was associated with higher odds of being in contact with health services for youth (males: 1.5;1.1–2.1, females: 1.7;1.4–2.1), but not the school health service.

Among females, but not males, being a current smoker was associated with higher odds of depressive symptoms (1.7;1.4-2.0), daily headache (1.5;1.1–1.9), and high medication use (1.6;1.3–2.1). Being currently smoking was associated with higher odds of self-harm (males: 1.7;1.3–2.3, females: 2.3;1.9–2.8), suicidal thoughts (males: 1.4;1.1–1.7, females:2.0;1.7–2.4), and suicide attempts among females (2.8;2.1–3.9), but not males. Both smoking males and females had higher odds of contacting school health services (males: 1.4;1.1–1.7, females: 1.3;1.1–1.6), and smoking males, but not females, had higher odds of being in contact with health services for youth (2.0;1.5–2.7). Interaction analyses (Table S2) also indicated that for males and females, sexual abuse interacted with smoking in predicting suicidal thoughts. For females alone, sexual abuse also interacted with smoking in predicting suicide attempts.

Final analyses (Table S3) showed that male smokers exposed to sexual abuse had 6.0 times increased likelihood of suicidal thoughts, whereas non-smokers exposed to sexual abuse had 3.1 times increased likelihood of suicidal thoughts compared to males who had not been exposed to sexual abuse. Among females, current smokers exposed to sexual abuse had 2.6 times increased likelihood of suicidal thoughts and 3.0 times increased likelihood of suicide attempt, whereas non-smokers exposed to sexual abuse had 3.6 times increased likelihood of suicidal thoughts and 5.8 times increased likelihood of suicide attempt compared to those not exposed to sexual abuse.

## Discussion

Results from the present study showed that a much higher proportion of females had experienced sexual abuse than males (10.9% vs. 2.3%). In accordance with our results, a study found that nine females and three males out of 100 are childhood victims of forced intercourse worldwide [47], but due to differences in design and methodological diversities, most results based on single studies from the Nordic countries are not comparable [11, 12]. Despite heterogeneity between studies and lack of standardized measures, the variation in prevalence of sexual abuse according to age and sex has been confirmed by several studies across continents [8, 28, 29].

Male and female victims of sexual abuse have expressed a journey of deep silent suffering, and the majority is living with numerous and often complex health problems [33]. Results from the present study indicate strong associations between sexual abuse, and daily headache, high medication use, depressive symptoms, self-harm, suicidal thoughts and suicide attempts. A few studies have previously confirmed that traumatic experiences such as sexual abuse, was associated with higher risk of headaches in children [48] and tension headaches in adults who also reported to employ immature and neurotic defense styles as a response to stress compared to healthy individuals [49]. In addition to physical impact, the literature on sexual abuse in childhood and adolescence shows that this problem has long-term psychological, sexual and social impacts on the victims’ lives [1, 15]. Specifically, a systematic review and meta-analysis reported a 4.0-fold increase in suicide plans and a 3.4-fold increase in suicide attempts among young individuals exposed to sexual abuse, with higher rates of suicide attempts among those who were not under the care of clinicians [24].

Although results from the present study confirm that more females than males were victims of sexual abuse, more male than female victims consistently reported to suffer from a range of health-related problems and suicidal behavior. In line with our results, some studies have previously indicated that males are more vulnerable to the adverse effects of childhood sexual abuse on mental health and suicidal behavior [13, 50]. Differences in health-risk behaviors and health consequences among sexually abused adolescents have also been explained by differences in use of coping strategies: women have a tendency to internalize their emotional pain, whereas men have a tendency to externalize it [33]. Given our study’s focus on adolescents aged 16–19 years, the poorer health outcomes among boys exposed to sexual abuse may partly be explained with reference to masculinity ideals and perceptions of one’s own physical strength. Interviews of males exposed to sexual violence have also indicated that the source of suicidal thoughts after sexual violence is based on an experienced strong self-destruction force, and that perceived common societal norms about male masculinity prevented them from expressing emotional pain and disclosing their traumas [51]. As supported by a previous study of male victims of CSA [52], boys who have been exposed to sexual abuse may experience an additional burden if they believe that they were, or should have been, able to prevent the abuse by their own powers. For sexually abused females, results from a longitudinal study have suggested that interventions should target shame, self-blame, and avoidance coping to foster recovery and reduce poor mental health outcomes and suicide ideation [53].

Based on results from other studies showing an association between substance use, mental health problems and increased risk of self-harm and suicidal behavior among sexually abused adolescents [13, 23], our study included information on concurrent exposure to alcohol intoxication, smoking and cannabis use in the models. In general, our results indicated a significant association between substance use and adverse health outcomes and suicidality, but results varied according to sex and drug types. Whereas exposure to sexual abuse and cannabis use was more strongly associated with adverse health effects in males, smoking was more strongly associated with adverse health outcomes in females than males. Contrary to our results, worse mental health status has been reported in females exposed to CSA compared to males exposed to CSA, whereas no difference in cigarette use was reported among females and males exposed to CSA [54]. A systematic review, which reported a strong association between cannabis use and risk of negative mental health outcomes among adolescents exposed to sexual abuse, suggested that intervention studies should target adolescent cannabis use in order to mitigate these risks [55].

However, interaction analyses from the present study indicated that cannabis use, as well as alcohol intoxication, did not modify the association between exposure to sexual abuse and adverse health outcomes in males or females. On the other hand, smoking was more strongly associated with adverse health outcomes in females than males. Moreover, interaction analyses indicated that for males exposed to sexual abuse, smoking was associated with increased odds of having suicidal thoughts, whereas for females exposed to sexual abuse, smoking was associated with decreased odds of suicidal thoughts and suicide attempts. To our knowledge, no other studies have previously reported a moderating effect of substance use on the relationship between sexual abuse and suicidal behavior, and it is unclear why results from the present study indicate different effects according to sex. One possibility is that adolescent boys and girls have different levels of tolerance for smoking, and therefore experience different physiological responses to it. Girls, who have a lower average body weight than boys, may experience smoking as a relatively stronger stimulus, inducing pleasure and reducing stress and anxiety [56]. While boys who have been sexually abused may seek this very effect, they may be less inclined to obtain it due to higher tolerance for the substance. It is also possible that among boys, smoking may be considered part of a larger pattern of self-destructive behaviors potentially arising from the abuse [51] – thus, the detected negative effect of smoking among boys may be explained by extraneous factors not assessed in this study. These gender differences have also been confirmed in a study among young adults which showed that low income and smoking were associated with attempted suicide in men, while attempted suicide in women was associated with poor self-evaluated health, low educational attainment, and drug use [57].

Finally, results from the present study showed a positive association between sexual abuse exposure and substance use, and use of youth health services. Further, our results showed a stronger association between exposure to sexual abuse and use of youth health services among males compared to females. These results are in contrast to a French study showing that sexually abused girls were twice as likely to use health services for psychological problems than boys [13]. Previous studies have also shown that compared to males, females are more likely to have disclosed their sexual abuse to others [58, 59]. Moreover, a systematic review concluded that females were more likely to seek emotional support by disclosing to peers, whereas males were less likely to disclose the abuse altogether, but when males do disclose, they tend to do so for practical reasons, such as protection or accessing services [60]. In order to reach adolescents exposed to sexual abuse, it is important that youth health services have the knowledge and training needed to provide support to both male and female victims. A study among males who had experienced sexual abuse, however, showed that they experienced lack of knowledge, understanding of suicidal thoughts and appropriate support from healthcare professionals and felt that sexual violence survivors’ trauma history needed to be better explored within health care [51]. Thus, the need to pay more attention to male victims of sexual abuse in health-care services by improving management guidelines, training of attending physicians and a supportive service to male survivors is warranted [30].

### Strengths and weaknesses of the study

The present study included a large representative sample of Norwegian adolescents, which is a major strength. To our knowledge, this is also the first study that has provided information about the association between both sexual abuse and substance use, and a range of adverse outcomes. This knowledge can most likely contribute to the development of improved youth health services specifically targeting vulnerable children and adolescents. The cross-sectional design of the study and the use of self-report measures are limitations that should be mentioned. Previous studies have also emphasized that the use of single informants of sexual abuse incidents diminishes validity of research on victim outcomes [14]. Another limitation of the present study is that we do not have information about age of abuse onset, frequency, and severity of abuse. Moreover, results from the present study relied exclusively on adolescents’ retrospective reports which, depending on possible recovery processes, may have led to under- or over-reporting of sexual abuse. As the present study did not include adolescents not in education, employment or training (NEETs), it is also likely that the prevalence of CSA in the present study has been underestimated, given that previous studies have shown that NEETs have more health problems than their peers in school [61]. Moreover, being NEET has also been associated with increased health-risk behaviors [62], and young NEET girls in particular have been identified to be vulnerable to self-directed violence and violence from others [40]. The present study is also limited by the lack of information about participants` use of other available health services, such as the general practitioner (GP) or psychologists. Finally, the Covid-19 pandemic may have influenced the results. Thus, future studies reflecting time trends of CSA and the association between CSA, substance use, adverse health outcomes and use of youth health services should be conducted.

## Conclusion and implications

To our knowledge, this is the first study to examine the unique sex-specific effects of sexual abuse on a range of health-related outcomes in a large, nationally representative sample of adolescents. Moreover, the present study provides valuable knowledge about the use of youth health services for adolescents exposed to sexual abuse and frequent substance use. Future studies should evaluate a broader set of abuse characteristics and include information about the number of abuse incidences utilizing a longitudinal design. Moreover, qualitative studies should explore possible explanations for large differences in health-related outcomes and use of youth health services among males and females exposed to sexual abuse. This study found that boys who had experienced CSA were comparably more likely than girls to seek relevant health services. As previous studies have suggested that boys may be particularly reluctant to seek appropriate help, future studies may explore the circumstances under which boys are more or less inclined to seek help. Thus, understanding sex-specific risk factors, signs, symptoms, and barriers to disclose and seek support after being exposed to sexual abuse can help to improve youth health services for adolescents by developing targeted programs that successfully identify, prevent, and treat this vulnerable population group.

## Electronic supplementary material

Below is the link to the electronic supplementary material.

**Supplementary material: Table S1**. Modeled effects of sexual abuse and substance use on depressive symptoms, daily headache and high medication use with interaction analyses. **Table S2**. Modeled effects of sexual abuse and substance use on self-harm, suicidal thoughts and suicide attempt with interaction analyses. **Table S3**. Adjusted analysis of the moderation of smoking and sexual abuse on the odds of suicidal thoughts among boys and girls and suicide attempt among females. **Table S4**. Use of youth health services according to exposure to sexual abuse and substance use risk behavior.

## Data Availability

Legal responsibility for the Young Data survey is held by the NOVA research center of Norwegian Social Research, Oslo Metropolitan University. Thus, the NOVA research center is responsible for the data sets analyzed during the current study and are available upon reasonable request (ungdata@oslomet.no).
