# Supplementary material for: Associations between exposure to sexual abuse, substance use, adverse health outcomes, and use of youth health services among Norwegian adolescents
Source: BMC Public Health. 2023 Jul 11;23:1330. doi: 10.1186/s12889-023-16261-y (PMC10337211; doi:10.1186/s12889-023-16261-y)
Supplement: Supplementary file 1 — Supplementary material: Table S1. Modeled effects of sexual abuse and substance use on depressive symptoms, daily headache and high medication use with interaction analyses. Table S2. Modeled effects of sexual abuse and substance use on self-harm, suicidal thoughts and suicide attempt with interaction analyses. Table S3. Adjusted analysis of the moderation of smoking and sexual abuse on the odds of suicidal thoughts among boys and girls and suicide attempt among females. Table S4. Use of youth health services according to exposure to sexual abuse and substance use risk behavior. [file 12889_2023_16261_MOESM1_ESM.docx]

**Supplementary material**

**Table S1.** Modeled effects of sexual abuse and substance use on depressive symptoms, daily headache and high medication use with interaction analyses.

|  | | **Depressive symptoms** | | **Daily headache** | | **High medication use** | |  |
| --- | --- | --- | --- | --- | --- | --- | --- | --- |
|  |  | Males  *OR (95% CI)* | Females  *OR (95% CI)* | Males  *OR (95% CI)* | Females  *OR (95% CI)* | Males  *OR (95% CI)* | Females  *OR (95% CI)* |  |
| **Exposure variables** | Sexual abuse | 2.7 (1.3-5.3)** | 2.9 (2.3-3.7)*** | 2.7 (1.3-5.3)** | 2.0 (1.5-2.8)*** | 2.4 (0.7-7.9) | 2.1 (1.5-2.9)*** |  |
|  | Alcohol intoxication | 0.8 (0.6-1.1) | 1.0 (0.8-1.2) | 0.8 (0.6-1.1) | 1.1 (0.8-1.4) | 1.5 (1.0-2.4) | 1.1 (0.9-1.5) |  |
|  | Smoking | 1.2 (1.0-1.6) | 1.6 (1.3-2.0)*** | 1.2 (1.0-1.6) | 1.6 (1.2-2.1)** | 1.5 (1.0-2.4) | 1.7 (1.2-2.3)** |  |
|  | Cannabis use | 1.9 (1.5-2.5)*** | 1.6 (1.3-2.1)*** | 1.9 (1.5-2.5)*** | 1.3 (1.0-1.7) | 1.5 (1.0-2.4) | 1.2 (0.9-1.6) |  |
|  | Cannabis use*sexual abuse | 1.9 (0.8-4.6) | 0.8 (0.5-1.3) | 1.9 (0.8-4.6) |  | 1.5 (0.4-6.3) |  |  |
|  | Sexual abuse*smoking |  | 1.1 (0.7-1.7) |  | 0.8 (0.5-1.3) |  | 1.0 (0.6-1.6) |  |
| **Control variables** | FAS | 0.8 (0.7-0.8)*** | 0.8 (0.8-0.8)*** | 0.8 (0.7-0.8)*** | 0.9 (0.8-1.0)** | 0.9 (0.8-1.0) | 1.0 (0.9-1.1) |  |
|  | Age | 1.0 (0.9-1.2) | 1.0 (0.9-1.1) | 1.0 (0.9-1.2) | 0.9 (0.8-1.0)** | 0.8 (0.7-1.0) | 0.9 (0.8-1.0) |  |

Note: *p<0.05, ** p<0.01, ***p<0.001.

**Table S2.** Modeled effects of sexual abuse and substance use on self-harm, suicidal thoughts and suicide attempt with interaction analyses.

|  | | **Self-harm** | | **Suicidal thoughts** | | **Suicide attempts** | |  |
| --- | --- | --- | --- | --- | --- | --- | --- | --- |
|  |  | Males  *OR (95% CI)* | Females  *OR (95% CI)* | Males  *OR (95% CI)* | Females  *OR (95% CI)* | Males  *OR (95% CI)* | Females  *OR (95% CI)* |  |
| **Exposure variables** | Sexual abuse | 0.9 (0.2-5.7) | 3.5 (2.7-4.5)*** | 3.2 (1.7-6.3)*** | 3.2 (2.5-4.1)*** | 6.0 (2.2-15.8)*** | 4.7 (3.2-7.0)*** |  |
|  | Alcohol intoxication | 0.7 (0.5-0.9)** | 1.0 (0.8-1.2) | 0.9 (0.7-1.1) | 1.0 (0.9-1.3) | 0.8 (0.5-1.4) | 1.1 (0.8-1.6) |  |
|  | Smoking | 1.7 (1.3-2.3)*** | 2.4 (1.9-3.0)*** | 1.3 (1.0-1.6)* | 2.2 (1.8-2.7)*** | 1.4 (0.9-2.1) | 3.7 (2.5-5.5)*** |  |
|  | Cannabis use | 2.0 (1.5-2.6)*** | 1.9 (1.5-2.5)*** | 2.2 (1.8-2.7)*** | 1.8 (1.4-2.2)*** | 2.6 (1.6-4.3)*** | 1.8 (1.2-2.8)* |  |
|  | Alcohol intoxication*sexual abuse | 1.7 (0.3-10.5) |  |  |  |  |  |  |
|  | Cannabis use*sexual abuse | 3.0 (1.0-9.1) | 0.9 (0.5-1.4) | 0.4 (0.2-1.0) | 1.4 (0.8-2.3) | 2.0 (0.6-6.5) | 1.2 (0.6-2-3) |  |
|  | Sexual abuse*smoking | 1.5 (0.5-4.2) | 0.8 (0.5-1.3) | **2.6 (1.1-6.5)*** | **0.6 (0.4-1.0)*** |  | **0.5 (0.3-0.9)*** |  |
| **Control variables** | FAS | 0.8 (0.8-0.9)*** | 0.9 (0.8-0.9)*** | 0.9 (0.8-0.9)*** | 0.8 (0.8-0.9)*** | 0.8 (0.7-0.9)*** | 0.8 (0.7-0.9)*** |  |
|  | Age | 0.8 (0.7-0.9)** | 0.6 (0.6-0.7)*** | 0.9 (0.8-0.9)** | 0.7 (0.7-0.8)*** | 0.7 (0.5-0.9)** | 0.6 (0.5-0.7)*** |  |

Note: *p<0.05, ** p<0.01, ***p<0.001.

**Table S3.** Adjusted analysis of the moderation of smoking and sexual abuse on the odds of suicidal thoughts among boys and girls and suicide attempt among females.

| **Interaction Variables** | **Suicidal thoughts** | **Suicidal thoughts** | **Suicide attempt** |
| --- | --- | --- | --- |
|  | Males  *OR (95% CI)* | Females  *OR (95% CI)* | Females  *OR (95% CI)* |
| ***Non-smokers*** | |  |  |
| Not exposed to sexual abuse | 1 (ref) | 1 (ref) | 1 (ref) |
| Exposed to sexual abuse | 3.1 (1.8-5.5)*** | 3.6 (2.9-4.5)*** | 5.8 (4.0-8.3)*** |
| ***Current smokers*** | |  |  |
| Not exposed to sexual abuse | 1 (ref) | 1 (ref) | 1 (ref) |
| Exposed to sexual abuse | 6.0 (3.4-10.8)*** | 2.6 (1.9-3.7)**** | 3.0 (2.0-4.6)*** |

Note: *p<0.05, ** p<0.01, ***p<0.001.

**Table S4.** Use of youth health services according to exposure to sexual abuse and substance use risk behavior.

|  | | **School health service** | | **Health service for youth** | |  |
| --- | --- | --- | --- | --- | --- | --- |
|  |  | Boys  *OR (95% CI)* | Girls  *OR (95% CI)* | Boys  *OR (95% CI)* | Girls  *OR (95% CI)* |  |
| **Exposure variables** | Sexual abuse | 5.7 (3.2-10.1)*** | 1.5 (0.9-2.5) | 14.9 (4.5-49.7)*** | 1.7 (1.0-2.9) |  |
|  | Alcohol intoxication | 1.2 (0.9-1.5) | 1.5 (1.3-1.8)*** | 2.0 (1.5-2.8)*** | 2.2 (1.8-2.6)*** |  |
|  | Smoking | 1.5 (1.2-1.9)*** | 1.5 (1.2-1.8)*** | 1.9 (1.4-2.6)*** | 1.2 (1.0-1.5)* |  |
|  | Cannabis use | 1.1 (0.8-1.3) | 1.2 (1.0-1.5) | 1.5 (1.1-2.1)* | 1.9 (1.5-2.5)*** |  |
|  | Alcohol intoxication*sexual abuse |  | 1.2 (0.7-2.1) | 0.4 (0.1-1.7) | 1.6 (0.9-2.8) |  |
|  | Cannabis use*sexual abuse |  |  | 0.6 (0.2-1.7) | 0.7 (0.5-1.2) |  |
|  | Sexual abuse*smoking | 0.5 (0.2-1.1) | 0.8 (0.5-1.3) | 0.7 (0.3-1.9) |  |  |
| **Control variables** | FAS (low) | 0.9 (0.9-1.0)** | 1.0 (1.0-1.0) | 0.9 (0.8-0.9)*** | 1.0 (0.9-1.0) |  |
|  | Age | 1.0 (0.9-1.1) | 1.0 (1.0-1.1) | 0.9 (0.7-1.0) | 1.1 (1.0-1.2) |  |

Note: *p<0.05- ** p<0.01- ***p<0.001.
